# Pre‐Treatment Specialist Interventions Improve Parents' Self‐Efficacy and Their Children's Eating Disorder Symptomology Before Commencing Outpatient Treatment

**DOI:** 10.1002/erv.3211

**Published:** 2025-05-24

**Authors:** Morgan J. Sidari, Daniel Wilson, Salvatore Catania, Victoria Brown, Edith Nkwenty, Amy Davis, Penny Knight, Jacinda White, Sarah Maguire, Tania Withington

**Affiliations:** ^1^ Queensland Eating Disorder Service Metro North Mental Health and Hospital Service Queensland Health Herston Australia; ^2^ Faculty of Medicine and Health InsideOut Institute University of Sydney Sydney Australia; ^3^ MAINSTREAM Centre for Health System Research & Translation in Eating Disorders Sydney Australia; ^4^ Child and Youth Mental Health Service Eating Disorder Program Children's Hospital Queensland Greenslopes Australia; ^5^ Child Health Research Centre University of Queensland Brisbane Australia

**Keywords:** adolescent, anorexia nervosa, parental support, psychoeducation, waitlist

## Abstract

**Background:**

Despite the severe and increasing burden of eating disorders (EDs) on children and adolescents, treatment services are typically only accessible after substantial waiting times. One strategy used to support families during the waitlist period is psychoeducation.

**Objective:**

This pilot study aimed to evaluate the effectiveness of a 6‐week pre‐treatment psychoeducation and specialist medical management and group‐based support programme for parents, targeting parental self‐efficacy and preparedness for their child's upcoming treatment at an Australian specialist outpatient ED clinic.

**Method:**

Waitlisted young people (197, mean age 14.55, SD = 1.70, 67% Anorexia Nervosa) and their parents (304) completed questionnaires assessing parents’ depression, anxiety and self‐efficacy, and young people's depression, ED symptomology and BMI; these measures were completed pre‐ and post‐programme and changes were assessed using multilevel models.

**Results:**

Parents increased in self‐efficacy, showed modest improvements in depression, and no significant change in anxiety. Young people showed significant improvements in global ED symptomology, BMI and depression.

**Conclusions:**

Our findings suggest that a pre‐treatment programme yields significant improvements in parental self‐efficacy, which is key to effective treatment of EDs in young people. Additionally, modest but meaningful improvements to young people's weight and psychopathology are achievable before families commence an evidence‐based outpatient treatment.

**Summary**

- Parental self‐efficacy increased following a 6‐week pre‐treatment programme for parents of young people waitlisted for outpatient ED treatment
- Improvements were also observed in young people's depression, global eating disorder symptomology and BMI
- A pre‐treatment programme involving psychoeducation plus specialist medical management and support was associated with meaningful gains for both parents and young people prior to commencing outpatient eating disorder specific therapy

## 1 Introduction

Despite the severe and increasing burden of eating disorders on children and adolescents and their families, treatment services are typically only accessible after substantial wait times. Given the overwhelming demand for treatment and significant resource limitations placed on health services, meaningfully reducing wait time for outpatient treatment is not always possible in practice (Devoe et al. 2023). Timely intervention is of the utmost importance in treating eating disorders, particularly in young people for whom adverse effects on growth and pubertal development can potentially be avoided or reversed with weight restoration (Neale et al. 2020). Additionally, increased wait time has been shown to predict dropout from outpatient services (O. Carter et al. 2012) and longer duration of symptoms has been associated with lower likelihood of recovery in adolescents (Forman et al. 2011) and adults (Von Holle et al. 2008). Timely treatment that facilitates early symptom improvement is a positive predictor of recovery. For example, early weight gain predicts remission during family based treatment (FBT; Le Grange et al. 2014) and early changes in dietary restraint and eating concerns predicts end of treatment reductions in global eating disorder psychopathology during enhanced Cognitive Behaviour Therapy (CBT‐E; Turner et al. 2015).

To address this gap in service provision, there is a need for evidence‐based, practical, scalable pre‐treatment programs (Radunz et al. 2022). Ideally, these pre‐treatment programs would improve young people's nutritional status and other key eating disorder metrics; however, at a minimum, maintaining their pre‐waitlist metrics would be preferable over the possibility of further decline while waiting to commence outpatient FBT (Lock and Le Grange 2012) or CBT‐E (Dalle Grave and Calugi 2020).

One strategy that has been used to support families during the waiting period is psychoeducation. Psychoeducation refers to interventions that offer individuals and/or their supports information regarding the disorder they are experiencing to promote understanding and adherence to treatment (Donker et al. 2009). Whilst not a stand‐alone therapy method, psychoeducation is likely a key element of behavioural therapy, and psychoeducation programs have shown promise as a pre‐treatment intervention across several mental health domains. In adults, psychoeducation programs have been shown to improve mental health symptoms and reduce drop‐out rates (Koksvik et al. 2018). Additionally, psychoeducation may facilitate early reductions in eating disorder symptoms prior to adult specialist treatments (Fursland et al. 2018); however, there is some evidence that these effects vary according to diagnosis, with effects noted for Bulimia Nervosa but not Anorexia Nervosa (Tatham et al. 2016).

For parents, psychoeducation programs have been shown to improve understanding of other mental health conditions that affect children and can result in greater alignment between families and treatment services/models and associated gains in wellbeing and symptomology. For example, for children with attention deficit hyperactive disorder (ADHD), a parental psychoeducation programme demonstrated significant improvements in their children's medication adherence and clinical ADHD symptoms (Yang et al. 2015). For children with learning disorders, a parental psychoeducation programme resulted in reduced criticism and increased warmth towards their children (Uslu et al. 2006). For children with anxiety disorders, a parental psychoeducation programme reduced children's anxiety levels and parents' parenting related stress; furthermore, this study found that psychoeducation was as effective as CBT in these domains (Lebowitz et al. 2020).

For mental health conditions, particularly those that are poorly understood and/or stigmatised, these programs may offer an opportunity to educate and empower parents who are feeling significantly overwhelmed and distressed. In line with the values of FBT, this empowerment of parents—termed parental self‐efficacy—is a critical component of young people's recovery from an eating disorder (Lock and Le Grange 2012). Importantly, parental self‐efficacy has been identified as a predictor of positive adolescent outcomes in eating disorder treatment (Robinson et al. 2013). Spettigue et al. (2015) used parental psychoeducation as a pre‐treatment programme targeting parents' self‐efficacy in treating the eating disorder. Significant increases in parental knowledge and self‐efficacy were reported; however, this study did not assess the impact on young people as only baseline measurements were used. To our knowledge, there have been no studies investigating both parent and young people's outcomes following group‐based pre‐treatment psychoeducation programs for eating disorders, despite early intervention being the top research priority identified by ED clinicians, consumers, carers and affiliates (Hart and Wade 2020).

Whilst promising results within eating disorders and across other mental health contexts suggest that parental psychoeducation programs are beneficial for parents and young people (e.g., Spettigue et al. 2015; Uslu et al. 2006; Yang et al. 2015), the large sample sizes required to precisely estimate the impact of these programs are often inaccessible for any one study in a community health setting. Given the operational time and resources required to develop and run pre‐treatment programs, quantifying the impact of these programs could give confidence to service providers, funders, and other stakeholders (Radunz et al. 2022). For this reason, we seek to contribute to a body of studies that will robustly evaluate the effectiveness of pre‐treatment programs for parents and their young people during the waitlist period for outpatient treatment.

The pre‐treatment programme, *Strong Foundations,* was introduced as a waitlist management strategy in the Child and Youth Mental Health Service Eating Disorder Programme at the Children's Hospital Queensland (CYMHS EDP) in 2021. The programme is designed to be transdiagnostic, intending to be suitable for all eating disorder diagnoses. The programme runs continuously on a 6‐week cycle and involves group psychoeducation sessions (parent‐only, once per week, online) and specialist medical management and support (young person and parent/s, once per week, face‐to‐face). The psychoeducation sessions are facilitated by allied health, nursing, psychiatry, and lived experience using a combination of didactic and interactive modes (see Caldwell et al. 2023; and Method below) and specialist medical management and support appointments are facilitated by nursing and psychiatry (see Method).

In this pilot study, we evaluated the Strong Foundations intervention. The primary outcome variables for the intervention were: parental self‐efficacy; and young person BMI centile and ED psychopathology. It was hypothesised that from pre‐ to post‐engagement with the Strong Foundations programme, parents would improve in self‐efficacy, and young people would improve in BMI centile and ED psychopathology. Secondary outcome measures were also included in the pilot study. For young people, depressive symptoms were evaluated across the programme as an additional measure of associated impairment likely secondary to ED symptoms. For parents, depressive and anxiety symptoms were included as a secondary outcome measure for exploratory purposes for two main reasons: (1) parental mental health psychopathology and outcomes are not well studied in the ED literature despite the central role of parents in the most well‐established treatment for adolescent ED (i.e., FBT); and (2) parental depression and anxiety may impact treatment outcome owing the relationship between these variables and well‐established predictors of outcome in FBT (i.e., parental self‐efficacy and expressed emotion; Allan et al. 2018; Elgar et al. 2007; Lovejoy et al. 2000; Rhodes et al. 2005; Rienecke et al. 2016; Robinson et al. 2013). It was hypothesised that parental depression and anxiety would decrease from pre to post treatment.

## 2 Method

CYMHS EDP is a publicly funded specialist eating disorder service for children and adolescents (0–17 years) which has been described in Lim et al. (2023). The clinic developed the Strong Foundations programme as an evidenced‐informed pre‐treatment programme for parents of young people awaiting outpatient treatment for eating disorders (inclusive of all eating disorder diagnoses) with the primary goal to improve parents' understanding of eating disorders and ultimately improve parental self‐efficacy in treating the eating disorder (Caldwell et al. 2023).

Participation in Strong Foundations involves two main components: psychoeducation and specialist medical management and support. Parents were requested to attend the weekly group online psychoeducation sessions, and parents and young people were asked to attend weekly face‐to‐face specialist medical management and support appointments at the CYMHS EDP community clinic. Young people must also have attended their General Practitioner (i.e. family doctor) for medical monitoring of physical parameters, which are reviewed in the specialist medical management and support component.

### 2.1 Group Psychoeducation Sessions

The six weekly topics were (1) *general psychoeducation*, (2) *nutrition*, (3) *meal* *support*, (4) *distress tolerance and communication*, (5) *treatment of eating disorders*, and (6) *support services* (see Table 1 for further details). The format involved a didactic presentation delivered by a CYMHS EDP clinician, that was then followed by time for questions from the participants. Each session was delivered live over Microsoft Teams.

**TABLE 1:** Topics and broad descriptions for each weekly strong foundations session, also described in Caldwell et al. (2023).

	Topic	Contents
1	*Psychoeducation*	Types of eating disorders, warning signs, common behaviours, starvation syndrome, Minnesota starvation experiment, refeeding syndrome, medical monitoring, indicators for hospital admissions
2	*Nutrition in Recovery*	Dieting as a risk factor, nutrition in adolescence for growth and development, nutrition for re‐nourishing, nutrition considerations (dietary choices, food allergies/intolerances, nutrient deficiencies) for the individual and the family, physical changes during re‐nourishing, energy density in practice.
3	*Meal support*	Rationale for meal support, importance of re‐feeding, stages of meal support, helpful phrases, externalising the eating disorder, reflection activities
4	*Distress tolerance and communication*	Distress intolerance in eating disorders, communication techniques, emotion coaching (validation and provision of emotional and/or practical seats), arousal curves, emotion regulation techniques, breathing techniques, safety planning
5	*Treatment of eating disorders*	Medical monitoring components and importance, eating disorder myths, carer distress and support, types of therapies, FBT phases, CBT‐E stages, co‐morbidities, medications
6	*Carer Support Services and Resources (Butterfly Foundation, Eating Disorders Queensland (EDQ) and CYMHS EDP Consumer Carer Coordinator)*	Providing support and promoting hope for carers sharing recovery stories and useful carer support resources and information (online and in person), national helpline, recovery support organisations and services, workshops, support groups and other relevant programs.

The structure of the psychoeducation sessions was an open group that was designed to allow parents (or other caregivers) to join at any point within the cycle following a comprehensive mental health assessment and acceptance to the CYMHS EDP. Once treatment commenced, parents were no longer permitted to attend psychoeducation sessions, with the exception of the nutrition module, which remained accessible to all parents. If parents felt they would benefit from re‐attending a specific module, they could request permission from their allocated therapist.

### 2.2 Specialist Medical Management and Support Sessions

Specialist medical management and support sessions were attended face‐to‐face and required the presence of the young person and one or both of their parents. These sessions were managed by a Nurse Practitioner with support from Psychiatry Registrars and Senior Nurses. The Strong Foundations programme as whole was overseen by CYMHS EDP Medical Director (Psychiatry). The content of the specialist medical management and support appointments are described in Table 2.

**TABLE 2:** Topics and broad descriptions for each weekly strong foundations specialist medical management and support session.

Topic	Description
*Reviewing GP* *medical* *monitoring*—*Review of medical status*	Reviewing medical monitoring observations from the young person's GP to determine whether the young person is medically stable for community management. Repeat of physical examinations if indicated to determine the young person's medical stability for ongoing community management or to facilitate hospital admission. Medical stability is defined using the Queensland Child and Adolescent State‐wide guidelines for eating disorders. Facilitation of hospital admission occurs in consultation with general practitioner if required.
*Assessing and managing risks*	Complete MSE. Risk assessment and management includes suicidal ideation, deliberate self‐harm, aggressive behaviours and carer/parent fatigue.
*Medication review*	Review of prescribed medications or commence medication intervention if indicated to manage sleep, anxiety/low mood, distress and identified risk concerns.
*Providing containment*	Providing containment to distressed patients and families using validation, empathy, and distress tolerance techniques informed by FBT, CBT, and DBT principles.
*Supporting renourishment*	Providing FBT‐informed guidance and support for renourishment and weight restoration.
*Consultation and Liaison with relevant stakeholders*	Consultation and liaison with the young person's GP, other health professionals involved, school and work placement.

### 2.3 Materials

Participants completed a self‐report battery using iPads at an allocated time during their first appointment at the clinic. The primary outcome variables were assessed: for young people via the EDE‐A and BMI; and parents' self‐efficacy was measured using the PVA. Secondary outcome measures were measured via the PHQ9 (for young people's and parents' depression), and via the GAD7 (for parents' anxiety [GAD7]).

#### 2.3.1 Parents Versus Anorexia (PVA)

PVA is a seven‐item scale measuring parental self‐efficacy, which is defined as parents' self‐perceived ability to adopt a primary role in their child's eating disorder recovery (Rhodes et al. 2005). It has demonstrated acceptable reliability (*α* = 0.78) and has been shown to distinguish between parents who are waiting for treatment versus parents whose children have gained > 10% of their body weight as a result of treatment.

#### 2.3.2 Generalised Anxiety Disorder (GAD‐7)

GAD is a seven‐item scale designed to measure probable cases of generalised anxiety disorder in time limited settings. It has demonstrated excellent reliability (*α* = 0.95) and has been shown to correlate with Beck Anxiety Inventory and other measures of construct validity such as symptom‐related difficulties (Spitzer et al. 2006).

#### 2.3.3 Patient Health Questionnaire (PHQ‐9)

PHQ‐9 is the nine‐item depression module of the Patient Health Questionnaire, which is a self‐administered version of the PRIME‐MD diagnostic instrument for common mental disorders. It has demonstrated good reliability (*α* = 0.89) and has been shown to correlate with a mental health professional's assessments of patients' depression status (Kroenke et al. 2001).

#### 2.3.4 Eating Disorder Questionnaire Adolescent (EDE‐A)

EDE‐A is a version of the widely used Eating Disorder Examination Questionnaire (EDE‐Q; Fairburn and Beglin 1994), which was modified to be more developmentally appropriate for adolescents by (1) shortening reference time frames from 28 to 14 days and (2) simplifying language (J. C. Carter et al. 2001). For the purposes of this study, we have used the global score (comprised of the mean score of dietary restraint, eating concerns, weight concerns and shape concerns subscales) and refer to this score as ED symptomology.

#### 2.3.5 BMI Percentiles

Body‐mass index (BMI) was calculated as weight (kilograms) divided by height (meters) squared. In this study, weight and height were measured by clinicians, using calibrated scales and stadiometer in the clinic. BMI was then converted to age‐ and sex‐adjusted BMI percentiles based on CDC reference data using the R package ‘childsds’ (Vogel 2022). Please see Table 3 below for a full list of reporters for each measure included.

**TABLE 3:** List of the applicable measures and the individuals who reported them (or their constituent variables in the case of BMI percentile).

Measure	Parents	Young people	Clinician(s)
PVA (parental self‐efficacy)	X		
GAD‐7 (generalised anxiety)	X		
PHQ‐9 (depression)	X	X	
EDE‐A (ED symptomology)		X	
BMI (body‐mass index) percentile			X

### 2.4 Ethics

This project was granted ethical approval by Children's Health Queensland research ethics committee (HREC/20/QCHQ/67708).

### 2.5 Participants

Participants comprised of consecutive referrals to the CYMHS‐EDP from 04 March 2021 to 22 April 2023. Those who were suitable for treatment and consented were included in the study. Reasons for not being deemed suitable include (1) being over 18 years of age; (2) not meeting criteria for an eating disorder; (3) eating disorder not being the primary clinical concern; (4) not being willing/able to engage in treatment; (5) and being medically/psychologically compromised such that outpatient treatment was contraindicated. Eight participants were excluded from analyses as their caregivers did not state their relationship to the young person, which meant that we were unable to link their pre‐ and post‐programme scores.

Participants were 197 young people (91% female) and 304 caregivers (168 mothers, 124 fathers, 7 other; hereafter referred to as parents for simplicity). Participants' ages ranged from 9 to 17 (*M* = 14.55, SD = 1.70). Participants were most often diagnosed with anorexia nervosa (67%) or atypical anorexia nervosa (17%); however, as Strong Foundations is a transdiagnostic treatment, all eating disorder diagnoses were included in analyses. These included bulimia nervosa (4%), atypical bulimia nervosa (1%), binge eating disorder (< 1%), avoidant/restrictive food intake disorder (ARFID, 7%), and other specified feeding or eating disorder (OSFED, 4%).

Participants were assessed by a psychiatrist with experience in assessing and treating young people with eating disorders, and diagnosis applied according to ICD or DSM‐5 criteria following clinical evaluation with the young person and family, anthropometric and medical evaluation.

### 2.6 Statistical Analysis

R (version 4.2.1) and R Studio (version 2023.03.1 + 446) were used to conduct all analyses (Posit Team 2023; R Core Team 2009). Specialised packages were used for data wrangling (*dplyr*: Wickhametal. 2023), cleaning (*validate*: Van Der Loo and De Jonge 2021), scale/descriptive analyses (*psych*: Revelle 2023), multi‐level models (*lme4*: Bates et al. 2015; *lmerTest*: Kuznetsova et al. 2017), effect size calculations (*effectsize*: Ben‐Shachar et al. 2020) and graphing (*ggplot2*: H. Wickham 2016).

### 2.7 Missing Data

As the study design allows one or both parents to report at each time point, we opted to use multi‐level models (MLMs) to allow us to conserve as much of our sample as possible. MLMs can also handle missing data more generally without requiring imputations, assuming data is missing at random (MAR). To establish whether the assumption of MAR was appropriate, we performed a series of logistic regressions with outcome missingness at post‐treatment predicted by the pre‐treatment value. We found that families of young people with higher pre‐treatment global ED symptomology were *more* likely to be completers. Our result is consistent with MAR as missingness depends on an observed variable; however, it is of course not possible to rule out that the data is missing not at random (MNAR). Given that we found youth with more severe ED symptomology were over‐represented rather than under‐represented in our post‐programme sample, we considered the implications of this finding for our analyses. We posit that this may (1) limit generalisation of our results to youth with less severe ED symptomology, but also (2) strengthen our results by biasing estimates towards the effect that would be observed in a more severe subset of the ED population, which may underestimate our treatment effect.

### 2.8 Multilevel Models

For parent outcomes, MLMs include all young people who had at least one pre‐programme observation from one parent and post‐programme questionnaires from any number of parents (i.e. 0, 1 or 2). For parent models, MLMs include a fixed effect of *Time* and random effects of *Child* and *Parent* (nested within *Child*) in a random intercepts model. For child outcomes, MLMs include a fixed effect of *Time* and a random effect of *Child* in a random intercepts model. Random intercepts models account for ‘clustering’ in outcome variables based on their belonging to the same individuals or groups. To do this, it allows each group (e.g. each child, then each parent within a ‘child group’) to have its own intercept, thereby accounting for the differences in means between these groups. For all MLMs, we report unstandardised coefficients which can be interpreted as the expected change in the dependent variable's original units for a one unit increase in time (e.g. a coefficient of 0.10 for BMI percentile would reflect young people gaining 10 BMI percentile points from pre‐ to post‐programme). Additionally, for our sensitivity analyses, we report MLMs with a (dichotomous) interaction term based on the young person's BMI percentile falling below or above the applicable threshold.

### 2.9 Cohen's d Effect Sizes

**FIGURE 1:**
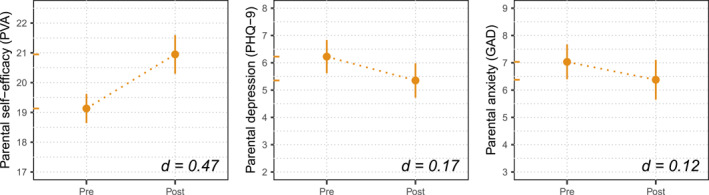
Points for pre‐ and post‐programme reflect the model's intercept (i.e. expected value at time 0) and intercept + estimate (i.e. expected value at time 1), respectively. Vertical bars depict the time 1 estimate's 95% confidence interval. The *Y*‐axis retains its original scale with limits set to ± 1 quartile to aid visual comparison across plots. For each model, both points are replicated as coloured tick marks on the *Y*‐axis. Cohen's d effect sizes are inlaid in the bottom right of the plot.

**FIGURE 2:**
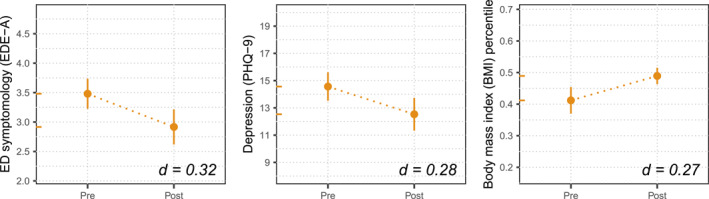
Points for pre‐ and post‐programme reflect the model's intercept (i.e. expected value at time 0) and intercept + estimate (i.e. expected value at time 1), respectively. Vertical bars depict the time 1 estimate's ± 95% confidence interval. The *Y*‐axis retains its original scale with limits set to ± 1 quartile to aid visual comparison across plots. For each model, both points are replicated as coloured tick marks on the *Y*‐axis. Cohen's d effect sizes are inlaid in the bottom right of the plot.

Cohen's d is reported based on the fixed effect of Time from each of our MLM analyses. For Cohen's d, we provide point estimates (inlaid in Figures 1 and 2) as confidence intervals for Cohen's d are poorly defined for multilevel models. We provide Cohen's d in line with convention; however, we suggest reviewing unstandardised estimates with sound clinical judgement.

## 3 Results

### 3.1 Baseline Characteristics

Means, standard deviations, and sample sizes are provided for age, height, weight and each of the dependent variables for young people (Table 4) and parents (Table 5) at pre‐ and post‐programme.

**TABLE 4:** Means and standard deviations for young people subdivided by diagnosis and time (pre, post).

	Age (years)			Height (cm)			Weight (kg)			BMI percentile			Depression (PHQ9)			ED symptoms (EDE‐A)		
Time	Mean	SD	*N*	Mean	SD	*N*	Mean	SD	*N*	Mean	SD	*N*	Mean	SD	*N*	Mean	SD	*N*
AN																		
Pre	14.65	1.58	122	164.27	8.01	122	50.16	8.54	122	0.35	0.26	122	14.17	6.85	127	3.53	1.68	129
Post	14.47	1.70	68	164.50	8.76	68	51.73	8.55	68	0.43	0.26	67	11.64	7.85	53	2.90	1.86	54
BN																		
Pre	14.12	1.46	8	168.96	8.57	8	61.72	8.83	8	0.71	0.14	8	17.71	6.13	7	4.68	0.77	7
Post	14.00	1.73	5	173.22	5.40	5	62.44	4.60	5	0.66	0.15	5	16.00	4.08	4	3.90	0.68	4
A‐AN																		
Pre	14.65	1.52	31	165.10	7.55	31	60.27	10.52	31	0.68	0.23	31	17.52	5.54	31	4.35	1.29	32
Post	14.65	1.37	17	166.18	6.32	17	65.77	10.34	16	0.79	0.15	16	16.47	5.89	15	4.09	1.26	16
A‐BN																		
Pre	16.00	NA	1	169.10	NA	1	61.85	NA	1	0.64	NA	1	24.00	NA	1	3.89	NA	1
ARFID																		
Pre	13.33	2.67	12	150.26	15.39	12	38.61	12.60	12	0.21	0.27	12	6.21	5.48	14	0.48	0.66	14
Post	12.83	3.06	6	143.93	14.90	6	36.74	11.57	6	0.33	0.35	6	4.00	4.18	5	0.44	0.83	5
OSFED																		
Pre	15.00	1.00	7	163.93	8.81	7	53.20	14.91	7	0.38	0.35	7	20.38	5.55	8	3.42	1.53	8
Post	15.00	NA	1	154.30	NA	1	41.45	NA	1	0.15	NA	1	18.00	0.00	2	3.45	0.00	2

*Note:* All diagnoses are based on ICD‐10 criteria. In line with this, young people with BMI > 17.5 can be diagnosed with anorexia nervosa provided that their weight is at least 15% below that expected (either lost or never achieved), with expected body weight calculated if premorbid data were available through medical records.

**TABLE 5:** Means and standard deviations for parents subdivided by time (pre, post).

Parents									
	Parental efficacy (PVA)			Anxiety (GAD7)			Depression (PHQ9)		
Time	Mean	SD	*N*	Mean	SD	*N*	Mean	SD	*N*
Pre	19.25	3.81	283	7.02	5.24	284	6.28	5.25	283
Post	20.80	3.92	132	6.29	5.35	131	5.02	4.93	131

### 3.2 Feasibility

Of the 197 young people who were assessed within the study time period, 81 young people (and 133 of their parents) provided end‐of‐study measures, resulting in a 42% retention rate. Unfortunately, the number of sessions families attended was not always recorded, which made families' engagement difficult to assess. For participants who did have their number of sessions recorded by a clinician at completion (46/81), the mean number of sessions attended was 3.97, suggesting that attending approximately 4 of 6 sessions may have been typical.

### 3.3 Multilevel Models

For all outcomes, graphs depict the unstandardised estimates (*b*) for *Time* from multilevel models. These visualise the expected change from pre‐ to post‐programme on the outcome's original scale.

### 3.4 Parent Outcomes

For parents, we observed a significant increase in parental self‐efficacy from pre‐ to post‐programme (*b* = 1.82, 95% CI = 1.17 to 2.46). For depression, we observed a statistically significant decrease from pre‐ to post‐programme (*b* = −0.87, 95% CI = −1.50 to −0.25); however, the effect size for this change suggests that it was very modest. For anxiety, we did not observe significant change from pre‐ to post‐programme (*b* = −0.65, 95% CI = −1.38 to 0.07). Results are shown in Figure 1.

### 3.5 Youth Outcomes

For young people, we observed significant improvements for all outcomes from pre‐ to post‐programme, as shown in Figure 2. Specifically, we observed a decrease in ED symptomology (*b* = −0.56, 95% CI = −0.86 to −0.27), a decrease in depression (*b* = −2.03, 95% CI = −3.22 to −0.84) and an increase in BMI percentile (*b* = 0.08, 95% CI = 0.05 to 0.10).

### 3.6 Sensitivity Analyses

Given the mean BMI percentile in our overall sample was higher than in some other outpatient settings, we ran two sensitivity analyses. For each young person and parent outcome, we ran one additional model with an interaction term for (1) BMI below the 5th percentile (Zanna et al. 2021) and one for (2) BMI below the 25th percentile (Le Grange et al. 2020). The sample size for the analysis included all participants for whom BMI/their child's BMI data was available at pre‐programme (178 young people, 285 parents). We found no evidence of moderation for any outcome variable under either of the two additional models (*p* > 0.087).

## 4 Discussion

In this pilot study, we aimed to evaluate the effectiveness of Strong Foundations, and explore its effects on both the parents and young people, particularly in the domain of parental self‐efficacy, but also in parents' depression and anxiety and young people's depression, eating disorder symptomology (dietary restraint, eating/weight/shape concerns) and BMI.

As predicted, we observed significant increases in parental self‐efficacy. Our finding supports Spettigue et al. (2015), who also observed significant improvements in parental self‐efficacy following pre‐treatment psychoeducation. The majority of the young people in their sample were diagnosed with eating disorder not otherwise specified (EDNOS), whereas our sample was predominantly young people diagnosed with anorexia nervosa (67%). In this way, our finding extends theirs to support the effectiveness of psychoeducation for parents of young people with other eating disorders. Given the established importance of parental self‐efficacy in adolescents' recovery (Robinson et al. 2013), this finding was particularly encouraging.

Contrary to hypotheses, there was no significant change in parents' anxiety symptoms from pre‐ to post‐programme. Although the psychoeducation sessions included one module addressing young people's distress tolerance, Strong Foundations was not designed to directly target parental anxiety symptoms, which may require more focussed intervention to demonstrate change (e.g., Toussaint et al. 2020). In line with predictions, we found a detectable improvement in parental depression. However, the effect size suggests that this improvement was very modest. The programme's focus on psychoeducation (rather than parent psychopathology) and the short time frame of the intervention was unlikely to prove sufficient to meaningfully impact parental depression symptoms. Additionally, there may be a broader range of parental comorbid psychopathology that may explain why accessing ED treatment for their child was insufficient to improve depressive and anxiety symptomology further. As this study is part of a larger longitudinal project, it is our intention to further explore this in future.

Consistent with hypotheses, we observed reductions in young people's global eating disorder symptomology from pre‐ to post‐programme. Given that the young people were not directly exposed to skills or strategies aimed at addressing their ED symptoms, it may be that Strong Foundations indirectly influences these symptoms, which may be mediated through its effects on parents. It is possible that parents' exposure to common eating disorder symptoms and behaviours through the psychoeducation sessions or discussions with specialist clinicians could have led to greater ability to identify and circumvent these behaviours. Increased parental self‐efficacy could also have led to greater confidence and skill in discussing eating disorder related topics and supporting healthy behaviours/challenging eating disorder behaviours with their children. Future research could explore this hypothesis through a mediation model.

Consistent with our hypotheses and the reduction in young people's eating disorder symptomology, there was a significant increase in young people's BMI from pre‐ to post‐programme. Although the effect size for this change was small, halting further decline in nutritional status and medical stability while on the waitlist is critically important to reduce young people's risk of eating disorder sequelae (Neale et al. 2020). In this way, we believe that any detectable increase in BMI is clinically meaningful. Further, by only assessing weight gain and not change in weight trajectory from pre‐ to post‐programme, we may not have assessed the full impact of the programme on weight. Future research may consider investigating this further. It should be noted that the service in which this programme was piloted uses ICD‐10 diagnostic criteria, enabling identification of young people with AN who do not meet the DSM weight/BMI criteria but who are 15% or more below expected body weight (through weight loss or failure to achieve). Hence the higher average BMI in our AN sample—cases that under a DSM system would be diagnosed as atypical AN meet full‐criteria for AN under ICD‐10.

As predicted, there was a significant reduction in young people's depression symptoms from pre‐programme to post‐programme. It is possible that some of the skills (e.g. communication techniques, distress tolerance) that parents learnt during the psychoeducation sessions, or through discussions during specialist medical management and support appointments, changed the nature of their interactions with the young person. For example, they could have contributed to more positive and/or fewer negative interactions between parents and their child; the child may have felt more supported, and this may have had flow on effects for hopefulness and low mood. Previous research has shown that expressed emotion, particularly high emotional‐overinvolvement, significantly reduced from pre‐ to post‐intervention in a psychoeducation programme for relatives (Uehara et al. 2001). We may expect this to have a greater impact on the child's wellbeing than the parent, given that parents could adopt these techniques for the child's benefit, while still holding significant distress internally, which is supported by the larger effect size for reductions to young person as compared to parental depression. Another explanation for this could be that a portion of young people's depression symptoms are direct physical consequences of their eating disorder. For example, the improved nutritional intake suggested by an increase in BMI could directly improve the fatigue, impaired concentration, sleep dysregulation, and appetite dysregulation that are assessed in the depression measure (PHQ items 4, 7, 3 and 5, respectively). Of course, these two explanations are not mutually exclusive.

The results as discussed above associate the specific ED focussed elements of Strong Foundations as potentially being responsible for symptom reduction (e.g., increased parental knowledge leads to increased self‐efficacy leads to increased ability to circumvent ED behaviours in their children, which lead to improvements in weight outcomes, eating disorder and depressive psychopathology). An alternate explanation is that rather than any specific element of Strong Foundations contributing to change, it could be the symptom improvement is a result of: general attention associated with the intervention; placebo effects associated with expectancy (especially given the specialist nature of the ED clinic); and/or common factors (i.e., through therapeutic alliance).

### 4.1 Implications

Strong Foundations demonstrates an effective method for utilising the full multidisciplinary team to provide a timely and supportive start to engagement with treatment services. Our findings suggest that such a pre‐treatment programme yields significant improvements in parental self‐efficacy, which is key to effective treatment of eating disorders in young people. Additionally, our findings suggest that modest but meaningful improvements to young people's weight and mental health metrics are achievable before young people and their families commence FBT or CBT. This is important, as early symptom change is the strongest predictor of treatment outcome (Vall and Wade 2015). If this can begin prior to beginning treatment proper, this may enhance treatment outcomes through improved momentum at the start of treatment. While further research is needed to precisely quantify programs' impact relative to time/monetary investment, our findings suggest that implementing programs similar to Strong Foundations could be beneficial for services that are contending with long waitlists. Strong Foundations has now been adopted as the first treatment for all families accepted in the Eating Disorders Programme for Children's Health Queensland.

### 4.2 Strengths and Limitations

A strength of our study was the larger sample size in comparison to previous research. Relatedly, our use of multilevel models allowed us to use all observations and robustly evaluate the impact of the programme. Another strength was that we were able to assess pre‐ to post‐programme changes for both young people and their parents, thereby building on the promising findings presented by Spettigue et al. (2015), who were unable to assess changes in eating disorder psychopathology directly. A limitation of our study was that our sample mostly consisted of young people (and parents of young people) with anorexia nervosa and atypical anorexia nervosa (67% and 17%, respectively), which limits its generalisability to young people experiencing other eating disorders. Unfortunately, the subsamples of young people with disorders other than anorexia nervosa were too small to meaningfully test whether our programme's effectiveness was moderated by diagnosis (e.g. ARFID, 7%). Given anorexia nervosa has an earlier presentation (relative to BN) and greater public awareness (relative to ARFID and BED), it is likely that diagnosis proportions will be similar in future studies. Likewise, the cohort was predominantly female (91%), which limits generalisability to other genders. For this reason, future research could consider larger overall sample sizes to allow researchers to test for moderation by diagnosis/gender and work towards improving equity in the evidence base for eating disorder treatment. Some participants who were medically unstable upon assessment were referred straight to hospital as per the exclusion criteria, no data was collected for these participants, which biases our sample to miss some more severe cases. A further limitation of this study was the lack of control group, which undermined our ability to assess whether the observed pre‐ to post‐programme differences resulted from the programme, from the general therapeutic attention, or the passage of time. It is possible that acceptance into the programme may have given families some hope and momentum, which could theoretically have improved outcomes regardless of whether pre‐treatment was genuinely impactful. Even so, there would still appear to be utility to the programme given that longer symptom duration and wait time are generally associated with poorer outcomes (O. Carter et al. 2012; Forman et al. 2011).

## 5 Conclusion

Community mental health services have limited resources, meaning time and funding spent on new initiatives must be judiciously allocated to avoid impacting services' core functions. As waitlists for eating disorder treatment programs persist, evaluations of pre‐treatment programs as reported here may assist decision‐making for these services and their stakeholders. We found that Strong Foundations was a feasible pre‐treatment adjunct and was effective in improving parents' self‐efficacy prior to their child's eating disorder treatment. Importantly, we found that young people experienced improvements in their BMI, ED symptomology and depression while on the waiting list. Future research could examine the impact of pre‐treatment programs on retention in treatment and on overall treatment duration with a view of assessing whether duration may be shorter in those who attend pre‐treatment sessions. Future research could also investigate the most important elements of the programme, as well as including further elements not covered in the current format (e.g., improving motivation for change, addressing child/parent relationship). More generally, future research with a control group and sufficient sample size to test for moderation by diagnosis would assist in providing further confidence in these results. Once a suitable number of studies are published, future research could also include dismantling study or meta‐analyses to better understand the impact of different programme features (e.g. live vs. pre‐recorded) on programme effectiveness.

## Ethics Statement

The project was approved by the Children's Health Queensland Hospital and Health Service Human Research Ethics Committee (EC00175) HREC/20/QCHQ/67708.

## Data Availability

The data that support the findings of this study are available on request from the corresponding author. The data are not publicly available due to privacy or ethical restrictions.
